# Factors associated with higher falling risk in elderly diabetic patients with lacunar stroke

**DOI:** 10.1186/s12902-022-01122-3

**Published:** 2022-08-08

**Authors:** Jianlan Jin, Song Wen, Yanyan Li, Mingyue Zhou, Qingqing Duan, Ligang Zhou

**Affiliations:** 1 Department of Endocrinology, Shanghai Pudong Hospital, Fudan University, Shanghai, 201399 China; 2 Helen Driller Family Comprehensive Cancer Center, University of California, San Francisco, USA; 3 Department of Radiology, Shanghai Pudong Hospital, Fudan University, Shanghai, 201399 China; 4 Shanghai Key Laboratory of Vascular Lesions Regulation and Remodeling, Shanghai Pudong Hospital, Shanghai, 201399 China

**Keywords:** Type 2 diabetes, Fall risk, Lacunar infarction, Age, Microvascular diseases

## Abstract

**Purpose:**

The aim of this study is to explore the factors associated with the fall risk in type 2 diabetes (T2D) patients with a lacunar stroke.

**Materials and methods:**

We compiled data of 146 T2D patients (mean age 68 years), including the Morse fall scale data (MFS), nutrition score, self-care scale, laboratory data, and data from continuous glucose monitoring system (CGMS) from 2019 to 2021 in Shanghai Pudong Hospital. Thereby, we evaluated the associations between MFS and other clinical parameters.

**Results:**

The analyses showed that there were significantly increased size and numbers of lacunar infarction (*p* < 0.05). Furthermore, the greater risk group had an older mean age (*p* < 0.05), and significant decreased estimated glomerular filtration rate (eGFR), total triglyceride (TG), while increased microalbuminuria, magnesium, lipoprotein A (LP(a)), anti-thyroid peroxidase antibody (TPOAb) (*p* < 0.05). However, the time in range (TIR) was very comparable (*p* > 0.05). The correlational study revealed the higher score of MFS was associated with the age (*r* = 0.41), number of lacunar infarction (*r* = 0.18), nutrition score (*r* = 0.20), self-care score (*r* = − 0.43), serum creatine level (*r* = 0.19), eGFR (*r* = − 0.26) (*p* < 0.05). The total numbers of lacunar infarction were associated with age (*r* = 0.36), eGFR (*r* = − 0.40), homocysteine level (*r* = 0.33) (*p* < 0.05).

**Conclusions:**

Age, nutrition, self-care ability, and renal function are all critical factors associated with the risk of fall in T2D with lacunar infarction. The age, eGFR, and homocysteine are closely associated with lacunar infarction, suggesting that in T2D, evaluation of kidney dysfunction, homocysteine level in the elderly can predict lacunar infarcts and falls.

## Introduction

The risk of falling in elderly type 2 diabetic patients is a serious concern that should be assessed as soon as the patients are admitted to the hospital [1, 2]. The fall could start various cycles that affect the patient’s overall outcome [3]. Most fall complications include bone fracture, bedridden, infections, malnutrition, hypoactivity, coagulation disarrangement, and other expected fall-related consequences. Each year, one-third of persons over the age of 65 and one-half of adults over the age of 80 are predicted to fall worldwide [4]. Complications of microvascular diseases were frequently found in the health context among the multi-factors impacting the risk of falling. Lacunar infarction is most typically detected on magnetic resonance imaging (MRI), and it is thought to be caused by arteriosclerotic microvascular dysfunction in the subcortical brain. T2D plays a role in this pathological development. The implications of taking care of lacunar infarction are due to its higher association with subsequent episodes of stroke or cognitive impairment in T2D [5, 6], and the chronic global cerebral hypoperfusion, causing progressive cerebral atrophy, [7]. Indeed, half of the patients with a first-ever lacunar infarct have mild cognitive impairment of subcortical vascular features and its presence may be a predictor of subcortical vascular dementia in the medium-long term [8]. The majority of lacunar infarctions, on the other hand, were ignored clinically due to minimal symptoms and were only discovered by MRI a few years later. As a result, MRI screening is essential to detect potential lacunar infarction in T2D patients who do not have severe neurological symptoms [9]. Due to the conditions limiting the examinations of MRI in some T2D patients who have a high propensity to fall, the surrogate examination for evaluation may be necessary, thus in the present study, we analyzed the possible indices that had a higher correlation with the high risk of fall in T2D patients defined by a commonly used nursing scale: Morse Fall Scale (MFS) [10]. The present study also explored the association between MFS and other clinical parameters.

## Materials and methods

### Source of in-patient data

T2D Patients’ information was collected from the in-patient information system at Shanghai Pudong Hospital. We included 146 adult T2D patients with lacunar infarction from 2019 to 2021 in the Department of Endocrinology. The inclusion criteria of these patients were who had regular locomotor activity and an MRI scan that revealed the presence of cerebral lacunar infarction. The T2D diagnosis made using World Health Organization (WHO) guidelines published in 1999. The patients with type 1 diabetes mellitus (T1D), specific type of diabetes mellitus (DM), gestational DM, other secondary DM were excluded from the study. Other diseases, such as acute stroke, stroke with severe paralyze, arrhythmia, and temporary brain ischemia, severe infection were among the exclusion criteria. Severe diabetic conditions such as diabetic ketoacidosis, hyperglycemic hypersmolar state were also excluded.

### Ethical statement

The institution approved the publication of this research article of Shanghai Pudong Hospital, Fudan University. Informed written consent was obtained to publish this study and accompanying images.

### Collecting the data on the scores of Morse fall scale (MFS), nutrition score, self-care scale

The scale of MFS, nutrition score, self-care scale of each patient was evaluated by trained, employed nurses of our department. The MFS developed to identify patients at risk of falling consists of the following six items: (a) history of falling (0 = no, 25 = yes); (b) secondary diagnosis (0 = no, 15 = yes); (c) ambulatory aid (0 = bed rest/nurse assist, 15 = crutches/cane/walker, 30 = furniture); (d) intravenous (IV) or heparin lock (0 = no, 20 = yes); (e) gait (0 = normal/bed rest/immobile, 10 = weak, 20 = impaired); and (f) mental status (0 = oriented to own ability, 15 = forgets limitations).

The nutrition socre were determined using Nutritional Risk Screening Scale (NRS-2002), which includes 3 sections: (a) nutrition status (0 = None; 1 = mild, weight loss > 5% in 3 months or 50–75% of the normal food intake in the last week; 2 = moderate, weight loss > 5% in 2 months or BMI 18.5–20.5 kg/m2 and reduced general condition or 25–50% of the normal food intake in the last week; 3 = severe, weight loss > 5% in 1 month or BMI < 18.5 kg/m2 and reduced general condition or 0–25% of the normal food intake in the last week); (b) stress metabolism (severity of the disease) (0 = None; 1 = Mild stress metabolism: patient is mobile; increased protein requirement can be covered with oral nutrition; Hip fracture, chronic disease especially with complications e.g., liver cirrhosis, COPD, diabetes, cancer, chronic hemodialysis; 2 = Moderate: patient is bedridden due to illness; Highly increased protein requirement, may be covered with oral nutritions; Stroke, hematologic cancer, severe pneumonia, extended abdominal surgery; 3 = severe stress metabolism: patient is critically ill (intensive care unit); very strongly increased protein requirement can only be achieved with (par) enteral nutrition; APACHE-II > 10, bone marrow transplantation, head traumas); (c) age (0 = below 70 years; 1 = above 70 years). Total score were (a) + (b) + (c); ≥3 points: patient is at nutritional risk. Nutritional care plan should be set up; < 3 points: repeat screening weekly.

The self-care assessment adopted the scale of Barthel index assessment likert, which includes: (a) self intake ability(10 = total self care reserved; 5 = need some help; 0 = total dependent); (b) Bathing ability (5 = total self care reserved; 0 = dependent); (c) grooming ability (5 = total; 0 = dependent); (d) dressing ability (10 = total; 5 = need some help; 0 = dependent); (e) defecation ability (10 = can control; 5 = merely no incontinence; 0 = incontinence or coma); (f) urinary ability (10 = can control; 5 = merely no incontinence; 0 = incontinence or coma or catheterization); (g) self toilet ability (10 = total self care reserved; 5 = need some help; 0 = total dependent); (h) transfer ability (15 = total self-care reserved; 10 = need 1 person assist or instruction; 5 = need 2 persons help; 0 = total dependent); (i) walk ability (15 = total self-care reserved; 10 = need 1 person assist or instruction; 5 = in wheel chair; 0 = total dependent); (j) stair ability (10 = total self care reserved; 5 = need some help; 0 = total dependent); The total score is calculated by addition of the above mentioned score of each section, and self-care ability were categorized into severe (≤40), moderate (41–60), mild (61–99) and total self-care (100).

The standard scales of each patient were evaluated and completed on the day of patient arrived at the department based on the patient’s locomotor activity, nutrition status, and self-care function. Finally, the doctors were informed about each patient’s risk of falling to make the proper clinical decision. Patients were classified as high-risk or low-risk depending on whether their MFS score was greater than 45.

### Analyses on the data of continuous glycemic monitor system (CGMS), laboratory blood and urine tests, and MRI

The data of CGMS including time in range (TIR), time above range > 13.9 mmol/l (TAR> 13.9 mmol/l)，TAR> 10 mmol/l，time below range < 3.9 mmol/l (TBR < 3.9 mmol/l), and TBR < 3 mmol/l, were analyzed. Laboratory blood tests including hepatic biochemical tests, kidney function (eGFR, calculated via formula of CKD-EPI), electrolyte, insulin or c-peptide release tests, diabetic antibodies, indices related bone metabolism (i.e., N-terminal middle molecular fragment of osteocalcin (N-MID), 25 hydroxyl vitamin D3 (25-OH-VitD3)), thyroid function and urine tests including microalbuminuria tests were analyzed. Finally, a professional radiology physician examined the size and number of lacunar infarctions in MRI images, and the sizes were classified as 0-3 mm, 3-5 mm, 5-10 mm, 10-15 mm, and 15-20 mm based on the MRI imaging.

### Statistical analyses

Statistics analyses were performed in SPSS (IBM, version 26.0) and Prism (GraphPad, version 9.0). Two-way ANOVA was used to compare the distinction between the groups of high fall risk and low-risk in levels of laboratory tests and MRI lacunar infarction data. Spearman correlational analyses were performed to reveal the fall risks and lacunar infarction factors. Statistical significance was set at *p* < 0.05 level for all analyses.

## Results

### The baseline information, and the disparity of age, nutrition score, and self-care score between the group of high-risk and low-risk with lacunar infarction

Firstly, we measured the baseline characteristics of 146 patients. The mean age of patients was 68.34 ± 11.23 years; the proportion of male and female patients were 69 and 77, which was comparable; the mean BMI of patients was 25.699 ± 3.717 kg/m^2^. As for the glucose metabolic state, the mean FPG was 7.271 ± 2.357 mmol/L; the mean HbA1c was 9.068 ± 2.331%. The plasma lipids revealed that mean TC was 4.226 ± 1.068 mmol/L; the mean TG was 1.648 ± 0.940 mmol/L; the mean LDL was 2.627 ± 0.991 mmol/L; the mean HDL was 1.073 ± 0.305 mmol/L. We found that only age was significantly different between high-risk patients and low-risk patients (*p* = 0.001). However, there was no significant disparity on the nutrition score (*p* = 0.45) and self-care score (*p* = 0.79) (Table 1.).

**Table 1:** The baseline characteristics and the features on the lacunar measued in MRI in low or high risk patients

Characteristic	Total cohorts	Low risk cohorts	High risk cohorts	***p***-Value
**Patients (n)**	146	31	115	/
**Age (years)**	68.34 ± 11.23	62.32 ± 12.79	69.96 ± 10.24***	< 0.001
**Gender (M/F)**	69/77	14/17	55/60	0.792
**BMI (Kg/m**^**2**^**)**	25.70 ± 3.72	26.53 ± 4.24	25.32 ± 3.43	0.240
**FPG (mmol/l)**	7.27 ± 2.38	7.75 ± 2.68	7.11 ± 2.24	0.337
**TC (mmol/l)**	4.23 ± 1.07	4.46 ± 1.14	4.17 ± 1.05	0.215
**TG (mmol/l)**	1.65 ± 0.94	2.11 ± 1.12	1.53 ± 0.86**	0.006
**LDL (mmol/l)**	2.63 ± 0.99	2.73 ± 1.08	2.60 ± 0.97	0.558
**HDL (mmol/l)**	1.07 ± 0.31	1.04 ± 0.30	1.08 ± 0.31	0.606
**HbA1c (%)**	9.07 ± 2.33	9.19 ± 2.41	9.04 ± 2.32	0.766
**Nutrion assessment**	1.61 ± 0.77	1.52 ± 1.00	1.63 ± 0.71	0.45
**Self-care assessment**	94.69 ± 53.50	96.94 ± 9.19	94.09 ± 60.14	0.79
**Lacunar size (mm)**				
**0-3 mm**	2.23 ± 0.96	1.86 ± 1.22	2.33 ± 0.85*	0.047
**3-5 mm**	1.59 ± 2.16	0.43 ± 1.33	1.91 ± 2.24***	< 0.001
**5-10 mm**	3.99 ± 3.97	2.66 ± 3.75	4.35 ± 3.97*	0.034
**10-15 mm**	2.59 ± 5.15	1.14 ± 3.53	2.98 ± 5.45*	0.026
**15-20 mm**	0.84 ± 3.76	0.00 ± 0.00	1.07 ± 4.22**	0.008
**Lacunar numbers**				
**0.-3 mm**	9.09 ± 7.72	7.45 ± 7.85	9.53 ± 7.65	0.184
**3-5 mm**	1.53 ± 3.87	0.29 ± 1.01	1.870 ± 4.27***	< 0.001
**5-10 mm**	1.79 ± 2.35	0.84 ± 1.66	2.05 ± 2.45**	0.002
**10-15 mm**	0.47 ± 1.26	0.19 ± 0.65	0.55 ± 1.37*	0.043
**15-20 mm**	0.07 ± 0.33	0.00 ± 0.00	0.09 ± 0.36*	0.012
**Total numbers**	12.96 ± 10.89	8.77 ± 8.95	14.09 ± 11.12*	0.015

*Note*: *BMI* Body mass index; *FPG* Fasting plasma glucose; *TC* Total cholesterol; *TG* Total triglyceride; *LDL* Low-density lipoprotein; *HDL* High-density lipoprotein; *HbA1c* Glycosylated hemoglobin A1c. *: *p* < 0.05; **:*p* < 0.01; ***: *p* < 0.001

### The size and the numbers of lacunar infarction in MRI were increased in high-risk patients

Our analyses revealed that the size of the lacunar infarction was larger in the group of high-risk group when compared to the low-risk group (0-3 mm, *p* = 0.047; 3-5 mm, *p* < 0.001; 5-10 mm, *p* = 0.034; 10-15 mm, *p* = 0.026; 15-20 mm, *p* = 0.008;). We also noticed that the number of lacunar infarctions was significantly higher in the high-risk group, but not in the 0-3 mm size category (0-3 mm, *p* = 0.184; 3-5 mm: 1.870 ± 4.266 vs 0.29 ± 1.01, *p* < 0.001; 5-10 mm: 2.05 ± 2.445 vs 0.84 ± 1.66, *p* = 0.002; 10-15 mm, *p* = 0.043; 15-20 mm, *p* = 0.012; total numbers, *p* = 0.015) (Table 1).

### The CGMS indices were generally comparable between high-risk and low-risk groups

The CGMS indices including TIR, TAR> 10 mmol/l, TAR> 13.9 mmol/l, TBR < 3.9 mmol/l, TBR < 3.0 mmol/l showed no statistical significant (TIR, *p* = 0.73; TAR> 10 mmol/l, *p* = 0.84; TAR> 13.9 mmol/l, *p* = 0.98; TBR < 3.9 mmol/l, *p* = 0.54; TBR < 3.0 mmol/l, *p* = 0.41) (Table 2).

**Table 2:** Comparisons of CGMS indices and significant metabolic related factors in higher fall-risks between the high-risk and low-risk groups

	Total cohorts	Low risk cohorts	High risk cohorts	***p***-Value
**TIR %**	74.65 ± 20.77	73.490 ± 22.99	74.96 ± 20.23	0.73
**TAR > 13.9 mmol/L %**	0.05 ± 0.10	4.86 ± 8.17	4.92 ± 9.89	0.98
**TAR > 10 mmol/L %**	0.25 ± 0.20	25.68 ± 21.75	24.87 ± 19.49	0.84
**TBR < 3.9 mmol/L %**	0.01 ± 0.09	0.27 ± 0.78	1.39 ± 10.05	0.54
**TBR < 3 mmol/L %**	0.00029 ± 0.002	0.00 ± 0.01	0.04 ± 0.23	0.41
eGFR (ml/min*1.73m^**2**^**)**	86.49 ± 24.50	97.35 ± 22.75	83.65 ± 24.26*	0.02
**Microalbuminuria (mg/g)**	38.79 ± 50.24	20.69 ± 28.78	43.88 ± 53.80**	0.003
**Magnesium (mmol/L)**	0.79 ± 0.08	0.77 ± 0.05	0.80 ± 0.09*	0.02
**Lp(a) (mg/L)**	318.97 ± 312.65	168.95 ± 213.26	359.15 ± 324.12*	0.04
**TPOAb (IU/mL)**	113.23 ± 285.22	35.93 ± 9.58	134.45 ± 319.01**	0.002

*Note*: *TIR* Time in range; *TAR* Time above range; *TBR* Time below range. *eGFR* Estimated glomerular filtration rate; *LP(A)* Lipoprotein A; *TPOAb* Thyroid peroxidase antibody. *: *p* < 0.05; **:*p* < 0.01; ***: *p* < 0.001

### The comparisons of laboratory data between the high-risk group and low-risk group

We found, in the kidney function and regular urine tests, the eGFR (calculated via formula of CKD-EPI) was significantly reduced in high-risk group (*p* = 0.02); whereas the microalbumin (represented as albumin creatine ratio mg/g) increased distinctively (*p* = 0.003). The serum level of magnesium was also increased in high-risk group significantly (*p* = 0.02). In terms of plasma lipids and lipoproteins, we found the level of TG decreased substantially (*p* = 0.006); whereas that of LP(a) increased markedly (*p* = 0.04). Moreover, we found the level of TPOAb significantly increased in high-risk group (*p* = 0.002) (Table 2).

### Association of the Morse fall scale (MFS) with multi-factors including metabolic parameters

**Fig. 1:**
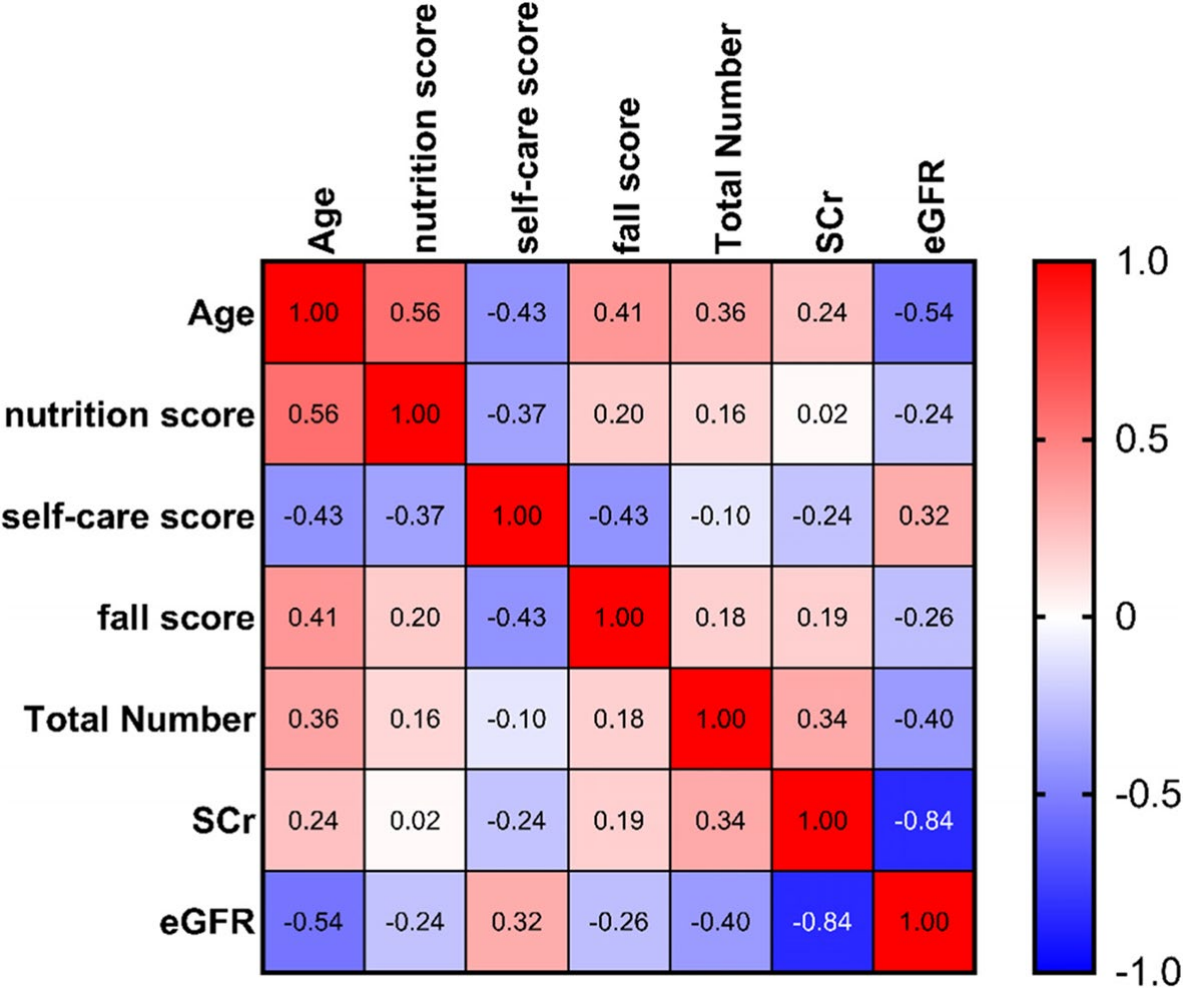
The heatmap shows the correlational relationship between the MFS and other parameters. The degree of the gradient in red represents the degree of positive correlation, and the blue represents the degree of negative correlation. Note: fall score: the score of Morse fall scale; Total number: the total numbers of lacunar infarction in MRI; SCr: serum creatine; eGFR: estimated glomerular filtration rate

We found in two groups the age (*r* = 0.41, *p* < 0.001), nutrition assessment score (*r* = 0.2, *p* = 0.02), self-care assessment score (*r* = − 0.43, *p* < 0.001), total numbers of lacunar infarction (*r* = 0.18, *p* = 0.03), the level of serum creatine (*r* = 0.19, *p* = 0.04), and eGFR (*r* = − 0.26, *p* = 0.007) were significantly correlated with the score of MFS (Fig. 1).

### Association of the metabolic parameters with the size and numbers of lacunar infarction in MRI

**Fig. 2:**
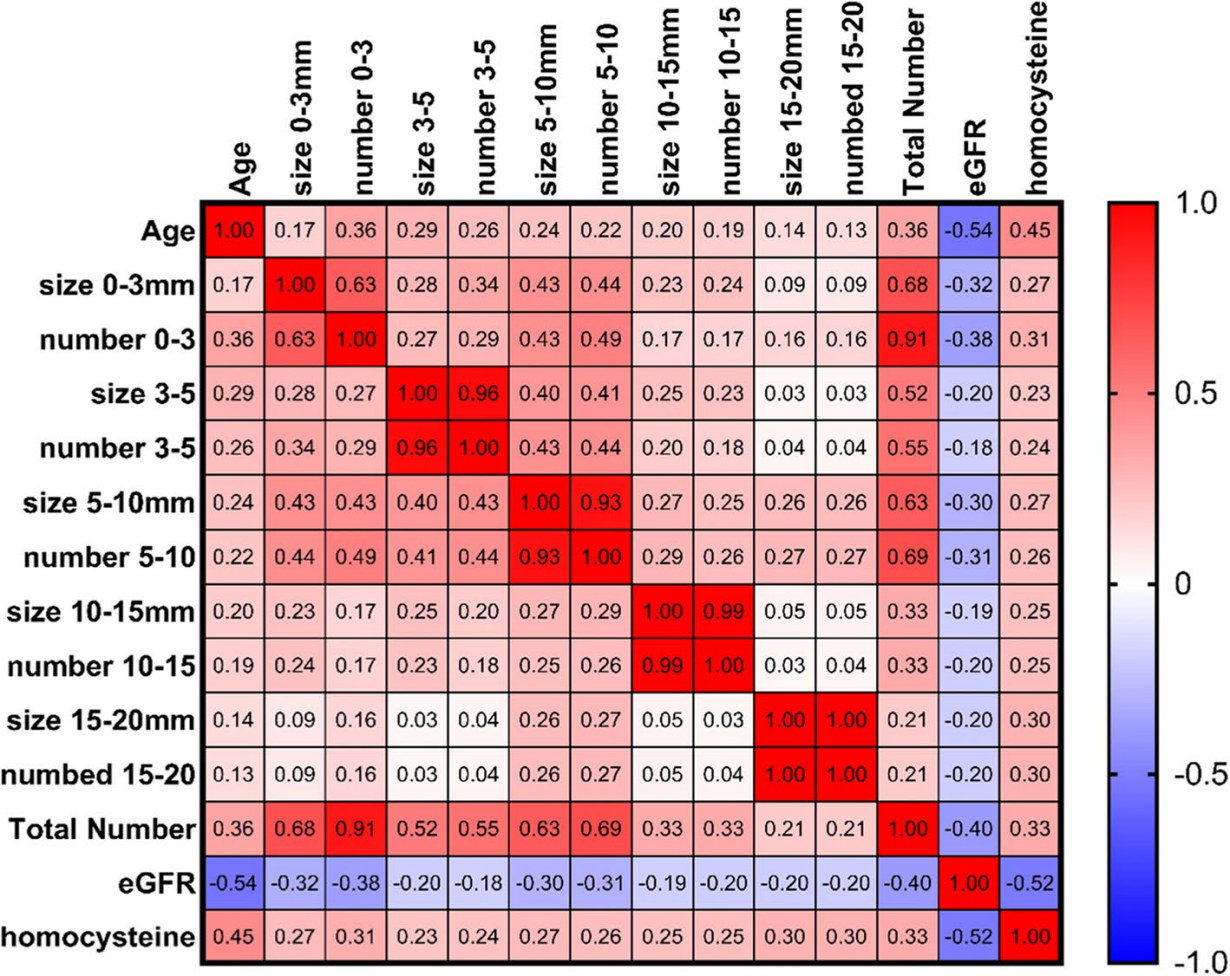
The heatmap shows the factors associated with the size and numbers of lacunar infarction: the age and level of the homocysteine were positively correlated whereas the eGFR was negatively correlated with the size and numbers of lacunar infarction

We further investigated the metabolic parameters linked to the size and quantity of lacunar infarctions in MRI. Unfortunately, the findings revealed that only age, eGFR, and homocysteine levels were substantially associated to the size and number of lacunar infarctions (total numbers: age: *r* = 0.36, *p* < 0.001; eGFR: *r* = − 0.4, *p* < 0.001; homocysteine: *r* = 0.33, *p* = 0.03) (Fig. 2).

## Discussion

In this study, we examined the factors contributing to a high risk of falling in T2D patients with lacunar infarction and found that age, the number of lacunar infarctions on MRI, SCR, eGFR, malnutrition, and self-care functions are all significant factors. First, we compared the disparity on metabolic parameters, nutrition score, and self-care score between high-risk and low-risk groups determined by MFS score. We only found ages were significantly older in the high-risk group (mean age 69.96 ± 10.24 years), demonstrating the tendency of a high incidence of falls in elderly T2DM patients due to deterioration of physical function [11]. However, we found no statistical differences in nutritional status and self-care scores between the two groups, suggesting the two groups are comparable in nutrition state and self-care function.

In the next step, we compared the size and numbers of lacunar infarction in MRI in two groups, and intriguingly, we found that all sizes and numbers of lacunar infarction were increased significantly in high-risk groups, suggesting the size and numbers of lacunar infarction is a critical factor that could lead to the fall in T2D patients. Lacunar infarction is one of the most critical MRI markers for diagnosing cerebral small vessel disease (CSVD), and when combined with other signs such as white matter hyperintensity (WMH), cerebral microbleeds (CMB), and enlarged perivascular spaces (EPVS), it can predict a higher risk of stroke in 5 years or more [12]. The CSVD belongs to the microvascular diseases, and its underlying pathological promoters include cerebral amyloid angiopathy (CAA) in ages exceed 55 years and atherosclerotic angiopathy with longstanding hypertension (HT) and diabetes mellitus (DM) [12–14] According to the clinical studies, half of the patients presented with initial lacunar infarction may influence cognitive function, which may be a predictor of subcortical vascular dementia in the medium-long term. Especially, mild neuropsychological disturbances (57.5%) are not infrequent in acute lacunar infarcts in patients with atypical lacunar syndrome and pure motor hemiparesis [8]. Therefore, the presence of cognitive impairment as the possible result of lacunar infarction could also be considered to be associated with an increased risk of falls.

In addition, we analyzed the glycemic state on admission to the department 3 days later by CGMS in two groups. However, we found the TIR was generally similar between the two groups, indicating that in the context of positive glucose control and preventing major hypoglycemia, the glucose level was not the indispensable predictor of the high-risk fall in T2D patients with lacunar infarction. Besides, we also did not figure out any disparity in both fasting and 2 hour-postprandial levels of c-peptide and insulin, and HbA1c. These findings may suggest that unless the major fluctuate in severe hyperglycemia or hypoglycemia, the propensity of fall is seemingly lower in T2D patients with lacunar infarction. Nonetheless, the glycemic level is critical to the micro- or macro-vascular pathological change [15]. Our findings suggested that glucose levels are not linked to the risk of falling at this loose glucose control level.

Furthermore, we compared renal function, regular urine tests, electrolytes, plasma lipids, lipoprotein, and thyroid function, all of which could play a vital role in the elevated risk of falling. We observed that eGFR was reduced, associated with increased microalbuminuria, indicating a diabetic-related microvascular change that could be an equivalent marker to CSVD pathological changes [16]. We also found TG was decreased whereas the LP(A) increased, which was consistent with the findings that LP(a) is a biomarker of cerebral cardiovascular disease [17]. We also found that TPOAb is significantly higher in the high-risk group. According to a recent study, the thyroid autoimmunity biomarker is related to metabolic syndrome in euthyroid patients [18]. All patients are in a euthyroid state, and we also discovered that TPOAb is much more significant in the high-risk group, indicating that the high-risk group is at a higher risk of metabolic syndrome [18, 19].

According to recent studies, in individuals with T2D, the risk factors for atherosclerosis macro-cardiovascular disease (macro-ASCVD) and atherosclerosis micro-cardiovascular disease (micro-ASCVD) may be partially distinct [20]. Combined lines of evidence suggest that LDL-cholesterol has a causal effect on the risk of peripheral arterial disease and chronic kidney disease (CKD), both of which represent manifestations of macro-ASCVD due to atherosclerosis and accumulation of LDL particles in the arterial wall. In contrast, there is limited evidence for a causal effect on the risk of microvascular disease. Hyperglycemia has a causal effect on the risk of micro-and macrovascular disease, and glucose-lowering may benefit the risk of micro-ASCVD and the risk of CKD and eGFR in studies [20, 21]. Thus, in present studies, we may find that in elderly T2D patients, the increased sizes and numbers of lacunar infarction may correspond with the reduced eGFR and increased microalbuminuria in high-risk groups.

Furthermore, we utilized Spearman’s correlational analysis to examine the relationship between the risk of falling and the factors mentioned above. The results revealed that age, nutrition status, self-care function, the total number of lacunar infarctions, SCr, and eGFR were all consistently related to the MFS score. These laboratory and imaging characteristics may indicate that strict hypoglycemia therapy should be avoided in clinical practice because of the risk of falls in older patients with brain and renal dysfunction. We also conducted a correlational analysis of the related factors in patients. We found that consistent with prior studies, the level of eGFR, which could be an equivalent biomarker of CSVD [22], could evaluate and forecast the risk of lacunar infarction or major stroke in T2D patients, along with homocysteine, a biomarker of cerebral cardiovascular diseases, could evaluate and forecast the risk of lacunar infarction or major stroke in T2D patients [23].

Our current research is limited by the data from the single-center. We gathered the data for the carotid artery from ultrasound, bone mineral density (BMD) exams, electromyography, and other sources. However, we did not quantify it or include it in the analyses because we believe they are incompatible with our goal of studying the effect of microvascular disorder and lacunar infarctions in the brain on the fall.

## Conclusion

The role of microvascular disease in T2D patients’ risk of falling, as measured by the MFS, was studied. According to our findings, age, nutritional status, self-care function, and microvascular abnormality as evaluated by eGFR and lacunar infarction were all connected to the risk of falling in older T2D patients. Even in glycemic well-controlled older T2D patients, evaluating the nutritional status, self-care capacity, and microvascular state could help predict fall risk and improve in-patient safety.

### Statement on guideline

The study was in accordance with related institutional guidelines on clinical study and regulations of human participants investigation of Shanghai Pudong Hospital, Fudan University.

## Data Availability

All data generated or analyzed during this study are included in this published article.
